# *H19*/miR-675 Axis Promotes Cancer Metastasis by Orchestrating EMT and MET Processes

**DOI:** 10.3390/cells15080658

**Published:** 2026-04-08

**Authors:** Kekely Klouyovo, Xuefen Le Bourhis, Éric Adriaenssens

**Affiliations:** University of Lille, Faculté des Sciences et Technologies, de Lille, Institut National de la Santé et de la Recherche Médicale (INSERM), CHU Lille, UMR9020-U1277-CANTHER-Cancer Heterogeneity Plasticity and Resistance to Therapies, ONCOLille Institute, 59000 Lille, France; kekely.klouyovo@gmail.com (K.K.); xuefen.le-bourhis@univ-lille.fr (X.L.B.)

**Keywords:** lncRNA, *H19*, miR-675, EMT, MET, cancers

## Abstract

**Highlights:**

**What are the main findings?**

- The H19/miR-675 axis is a key regulator of cell phenotype, coordinating EMT-driven dissemination and MET-associated colonization in cancer metastasis.
- H19 promotes EMT through multiple mechanisms beyond miRNA sponging, while miR-675 exerts context-dependent effects that can either support EMT or facilitate MET.

**What are the implication of the main finding?**

- The H19/miR-675 axis represents a dynamic regulatory node within complex metastatic networks rather than a linear pathway.
- This axis holds potential as a prognostic biomarker and therapeutic target, although its clinical application remains context-dependent and requires further validation.

**Abstract:**

Despite substantial advances in our understanding of cancer metastasis, it remains the leading cause of mortality among cancer patients. Elucidating the molecular mechanisms that drive metastatic progression is expected to facilitate the development of more effective therapeutic strategies. Among the numerous candidates, the long non-coding RNA *H19* and its derivative miR-675 have been increasingly recognized as key regulators of metastatic dissemination in cancers of diverse tissue origins. In this review, we provide an up-to-date overview of the *H19*/miR-675 axis in metastatic progression, with particular emphasis on its involvement in the dynamic and complementary processes of epithelial–mesenchymal transition (EMT) and mesenchymal–epithelial transition (MET). We also highlight the opportunity to consider the *H19*/miR-675 axis as promising biomarkers and potential therapeutic targets.

## 1. Introduction

Metastasis is a multistep process through which malignant cells disseminate from a primary tumor to establish secondary lesions in distant organs. It initiates with local invasion, during which cancer cells acquire the capacity to detach from the primary tumor, degrade the surrounding extracellular matrix, and infiltrate adjacent tissues. This is followed by intravasation, wherein tumor cells penetrate the walls of nearby blood or lymphatic vessels to enter the circulation. Once in the vasculature, circulating tumor cells (CTCs) must withstand hemodynamic shear forces, evade immune surveillance, and survive in an anchorage-independent manner. Only a small subset of CTCs successfully extravasates at distant sites by crossing the endothelial barrier. Upon colonization of a new microenvironment, cancer cells must adapt to local conditions, evade immune responses, and interact with tissue-specific components to establish metastatic lesions. Sustained growth and survival are further supported by the induction of angiogenesis, which ensures an adequate supply of oxygen and nutrients. Throughout this metastatic cascade, cancer cells exhibit remarkable phenotypic plasticity, dynamically transitioning between epithelial and mesenchymal states *via* epithelial-to-mesenchymal transition (EMT) and mesenchymal-to-epithelial transition (MET).

EMT is a dynamic biological process in which epithelial cells undergo phenotypic changes, acquiring mesenchymal traits that enhance migratory and invasive capabilities. During EMT, epithelial cells lose apico-basal polarity and cell–cell adhesion, primarily mediated by proteins such as E-cadherin (CDH1), ZO-1, and occludin, while upregulating mesenchymal markers, including N-cadherin (CDH2), fibronectin, vimentin, and MMP-2 [1,2]. These phenotypic changes are orchestrated by key transcription factors, including Snail, Slug, Twist, and ZEB1/2, which are activated through signaling pathways such as TGF-β, Wnt, and Notch [3]. EMT facilitates tumor dissemination by promoting motility, invasiveness, and resistance to anoikis. In addition, it is associated with the acquisition of stem cell-like properties and therapeutic resistance, thereby contributing to disease recurrence and metastatic progression.

MET, representing the opposite process of EMT, is characterized by the acquisition of a differentiated epithelial phenotype from a mesenchymal state, and the loss of motility. While EMT facilitates dissemination from the primary tumor by promoting motility, invasiveness, and resistance to apoptosis, MET is increasingly recognized as critical for the later stages of metastasis [4,5,6]. MET enables metastatic cells to integrate into host tissue architecture and support the establishment of proliferative metastatic outgrowths. Experimental models have shown that forced maintenance of a purely mesenchymal phenotype can impair metastatic colonization, underscoring the necessity of phenotypic plasticity [7,8].

EMT and MET processes are increasingly recognized to be regulated by non-coding RNAs, including long non-coding RNAs (lncRNAs) and microRNAs. *H19*, the first identified lncRNA, is frequently overexpressed in various cancers and has been implicated in promoting multiple cancer hallmarks, including therapy resistance and metastatic progression [9,10,11]. Several studies have reported that *H19* overexpression correlates with tumor progression and metastatic dissemination, particularly in solid tumors such as gastric and colorectal cancers. Its upregulation is associated with increased expression of EMT-inducing transcription factors and mesenchymal markers, underscoring a strong connection between *H19* expression, EMT signaling, and metastatic potential [12,13,14]. Similarly, analysis of a TCGA-SKCM melanoma cohort revealed that miR-675-3p is upregulated in metastatic tumors and linked to activation of the TGF-β pathway, further supporting its association with EMT signatures [15].

The *H19* gene is located at the position p15.5 on chromosome 11 and is subject to genomic imprinting. The transcript is capped, spliced and polyadenylated to give raise to a 2.3 kb RNA [16]. *H19* RNA can bind to other mRNA to stabilize their expression, interact with proteins or even act as a competitive endogenous RNA (ceRNA) to sequester microRNA and inhibit the binding to their targets, as reviewed in [17,18]. In addition to its direct effects, *H19* is also a precursor of the microRNA miR-675 [19], which has been increasingly reported to be involved in the regulation of both EMT and MET processes.

The aim of this review is to provide an up-to-date overview of the contribution of the *H19*/miR-675 axis to EMT-MET regulation and to discuss the potential clinical implications of targeting this pathway for the prevention of cancer metastasis.

The literature considered in this review spans from 1990 to 2025, with a primary focus on studies published in the last decade (2015–2025), reflecting the recent advances in the understanding of *H19*/miR-675 in EMT/MET plasticity. All articles were from PubMed and keywords included “*H19*”, “miR-675”, “EMT”, “MET” and “metastasis”. Articles were selected based on their relevance to the molecular mechanisms underlying *H19*/miR-675-mediated regulation of epithelial–mesenchymal plasticity.

## 2. Role of H19 in Promoting Metastasis Through EMT

The long non-coding RNA (lncRNA) *H19* has emerged as a key regulator of EMT, facilitating cancer cell invasion and dissemination during the initial stages of metastasis in multiple cancer types (Table 1). *H19* promotes EMT through diverse molecular mechanisms, including microRNA sponging, activation of oncogenic signaling pathways, and epigenetic and translational regulation.

### 2.1. MicroRNA Sponging

*H19* has been consistently reported to function as a competing endogenous RNA (ceRNA), promoting EMT across multiple cancer types (Table 1). Several microRNAs, including miR-200, let-7, miR-29, and miR-22-3p, have been increasingly recognized as mediators of *H19*-induced EMT in various cancers (Table 1).

The miR-200 family (miR-141, miR-200a, miR-200b, and miR-200c) is well established in maintaining the epithelial phenotype by targeting the EMT-inducing transcription factors ZEB1 and ZEB2. In multiple cancers, including glioma, breast, lung, esophageal, and colorectal cancers, *H19* overexpression promotes EMT and enhances tumor growth by sponging miR-200 family members, thereby relieving repression of ZEB1/2 [20,27,28,29,30,37].

**Figure 1:**
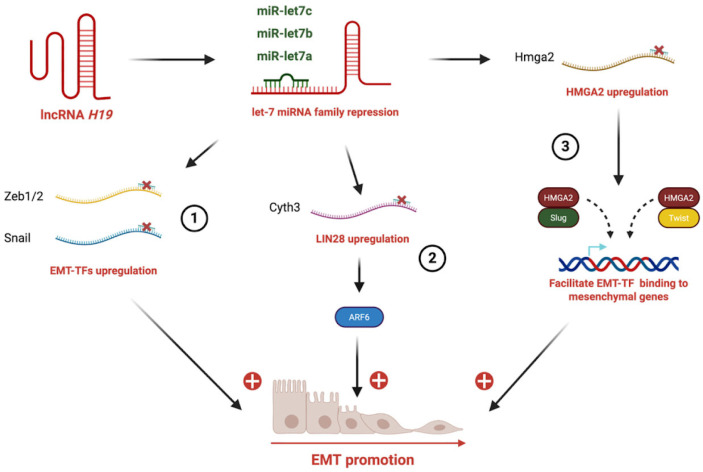
***H19* sponges let-7 miRNA to promote EMT through different mechanisms**. (1) *H19* sequestrates members of the let-7 miRNA family, leading to the upregulation of EMT-transcription factors (Zeb1/2, Snail). (2) Let-7 repression induces LIN28 upregulation and activation of its downstream effector ARF6, further reinforcing EMT programs. (3) Let-7 inhibition results in HMGA2 overexpression, HMGA2 in turn cooperates with mesenchymal transcription factors (Slug, Twist) to facilitate their binding to mesenchymal gene promoters.

The let-7 family (let-7a, let-7b, let-7c, etc.) has been implicated in *H19*-mediated EMT in breast, pancreatic, ovarian, and endometrial cancers (Table 1). Indeed, let-7 regulates the expression of multiple targets, including Lin28, Cyth3, HMGA2, and IL-6. For instance, in breast cancer, *H19*-mediated sequestration of let-7a prevents it from repressing Lin28, leading to elevated Lin28 expression [38]. LIN28 functions as a critical RNA-binding protein that represses the maturation of let-7 microRNAs. Suppression of the let-7 family results in derepression of its targets, including key EMT-inducing transcription factors such as ZEB1 and Snail, thereby enhancing cellular plasticity, migration, and metastatic potential [39]. On the other hand, *H19*-mediated sequestration of let-7b prevents repression of Cyth3, a guanine nucleotide exchange factor for ARF6 that regulates receptor trafficking and cytoskeletal remodeling, thereby promoting EMT and metastatic dissemination in breast cancer cells [29]. Another example has been shown in esophageal cancer, in which *H19* sponges let-7c, thereby relieving its inhibitory effect on STAT3. the resulting activation of STAT3 upregulates EZH2 expression, a component of PRC2 (Polycomb Repressive Complex 2) which is implied in chromatine modification [35]. In oral, pancreatic, ovarian, and endometrial cancers, *H19*-dependent sponging of let-7 leads to derepression of HMGA2 expression [34,36,40]. HMGA2 (High Mobility Group AT-hook 2), a non-histone chromatin-binding protein, is able to reshape chromatin, recruit coactivators, and facilitate binding of EMT TFs (Snail, Slug, Twist, ZEB1/2) to their target promoters [41]. Finally, *H19* sequesters let-7, derepressing IL-6 to facilitate EMT in cholangiocarcinoma (Figure 1) [33].

**Figure 2:**
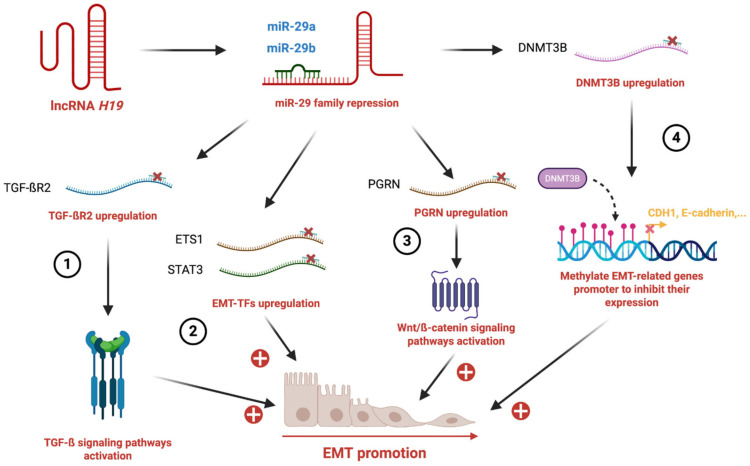
***H19* sponges the miR-29 axis to promote EMT.** (1) *H19* sponges miR-29 family members, leading to the upregulation of TGF-βR2 and activation of the TGF-β signaling pathway. (2) miR-29 repression results in increased expression of EMT-transcription factors (ETS1, STAT3), reinforcing EMT. (3) *H19* sponges miR-29 results in upregulation of progranulin (PGRN) which contributes to Wnt/β-catenin signaling activation. (4) *H19*-mediated miR-29 suppression leads to DNMT3B upregulation, which methylates promoters of epithelial genes such as CDH1 (E-cadherin), thereby repressing their expression.

miR-29 is another important microRNA family targeted by *H19* to promote EMT in several cancers such as breast, lung, colorectal and bladder cancers (Table 1). In breast cancer, *H19* interferes with miR-29a, leading to the de-repression of the transcription factor ETS1 [20]. ETS1 (E26 transformation-specific sequence 1), a member of the ETS family of transcription factors, promotes EMT and metastatic progression by inducing the expression of mesenchymal genes, including vimentin, N-cadherin, and matrix metalloproteinases (MMP-2 and MMP-9), as well as upregulating EMT-inducing transcription factors such as ZEB1 [42]. In lung cancer, *H19* sponging of miR-29b-3p, leads to the activation of STAT3 signaling and EMT [24]. In colorectal cancer, elevated *H19* levels sequester miR-29b-3p, thereby relieving miR-29b-3p-mediated repression of progranulin (PGRN, also known as GP88 or granulin–epithelin precursor). Increased PGRN activates the Wnt/β-catenin signaling pathway, leading to upregulation of β-catenin target genes, including c-Myc and cyclin D1, and promoting EMT [25]. In bladder cancer, sponging of miR-29b-3p induces the expression of DNMT3B (DNA methyltransferase 3B) which in turn enhances EMT and metastasis [26]. Indeed, DNMT3B, a de novo DNA methyltransferase, is involved in the establishment and maintenance of epigenetic patterns. In the context of EMT, DNMT3B contributes to the repression of epithelial genes, notably CDH1/E-cadherin, through promoter hypermethylation [43] (Figure 2).

Similar to the above-mentioned microRNAs, miR-29 is sequestered by *H19* to promote EMT in various cancers, including gastric and liver cancers (Table 1). In gastric cancer, aberrant *H19* overexpression sponges miR-22-3p, relieving its inhibitory effect on Snail1 and resulting in Snail1 upregulation [23]. In hepatitis B-related hepatocellular carcinoma (HBV-HCC), *H19* promotes tumor progression by negatively regulating miR-22 [21]. *H19* silencing reduces migration and invasion and reverses EMT, as evidenced by increased E-cadherin and decreased N-cadherin, vimentin, β-catenin, and MMP-9. Rescue experiments confirmed that inhibition of migration and invasion induced by *H19* knockdown could be reversed by miR-22 silencing, highlighting the role of the *H19*/miR-22 axis in EMT and metastasis in HBV-HCC. Finally, Zhu et al. demonstrated that miR-22 directly targets Twist1, leading to its downregulation and inhibiting EMT, proliferation and invasion in osteosarcoma cells [22].

Other microRNAs are also targeted by *H19* in EMT regulation. In paclitaxel-resistant breast cancer cells, *H19* promotes EMT via the *H19*/miR-340-3p/YWHAZ axis. *H19* functions as a competing endogenous RNA (ceRNA), sponging miR-340-3p and relieving repression of YWHAZ (14-3-3ζ), a regulator of cell survival, migration, and oncogenic transformation [32]. In hepatocellular carcinoma, *H19* promotes EMT by sequestering miR-326, thereby preventing repression of TWIST1, whereas *H19* knockdown suppresses EMT and inhibits proliferation and metastasis [31].

In ovarian cancer, *H19* sponges miR-370-3p, which normally represses EMT-related genes [44]. Upon TGF-β1 stimulation, *H19* expression increases while miR-370-3p declines, leading to upregulation of Snail and vimentin and downregulation of E-cadherin. Silencing *H19* or overexpressing miR-370-3p reverses these EMT features, confirming the functional relevance of the *H19*/miR-370-3p axis in TGF-β1-induced EMT.

Similarly, in a liver fibrosis model, *H19* acts as a ceRNA for miR-148a, derepressing USP4 (ubiquitin-specific protease 4), a miR-148a target involved in EndoMT regulation. The *H19*/miR-148a/USP4 axis promotes EndoMT by repressing E-cadherin [45]. Although observed in a precancerous context, this mechanism illustrates how *H19* can modulate USP4 availability to drive EMT.

### 2.2. Activation of Signaling Pathways

*H19* has been reported to promote EMT by activating the Wnt/β-catenin pathway in multiple cancers, including gastric, breast, and colorectal cancers. In gastric cancer, *H19* activates Wnt signaling by upregulating c-Myc and cyclin D1, promoting β-catenin nuclear translocation and induction of downstream targets such as Axin2, MYC, and TCF7 [46]. Similarly, in colorectal cancer, elevated *H19* promotes EMT through PGRN, which enhances expression of β-catenin target genes, including c-Myc and cyclin D1 [25]. In drug-resistant breast and colorectal cancer cells, *H19* is upregulated and drives EMT through Wnt/β-catenin activation, highlighting the *H19*-Wnt/β-catenin axis as a mediator of both EMT and drug resistance [47].

Beyond Wnt/β-catenin pathway, *H19* also regulates cell migration and invasion via NF-κB and PI3K/AKT signaling. In Helicobacter pylori-infected gastric cancer cells, *H19* enhances proliferation, migration, and invasion by augmenting NF-κB signaling, which can be reversed by pharmacological NF-κB inhibition [48]. In osteosarcoma, *H19* silencing reduces migratory and invasive capacities, concomitant with decreased PI3K/AKT phosphorylation and increased IκBα expression, indicating that *H19* promotes these behaviors by activating NF-κB through PI3K/AKT signaling [49].

*H19* not only activates oncogenic signaling pathways but is also regulated by them. Oncogenic factors such as HGF and EGF induce *H19* expression in mammary epithelial and breast cancer cells, respectively [50,51]. HGF-induced *H19* correlates with cell scattering, enhanced motility, and branching morphogenesis, while EGF-mediated *H19* upregulation stabilizes tyrosine kinase receptors, including EGFR and c-Met, by downregulating E3 ubiquitin ligases c-Cbl and Cbl-b. This stabilization leads to sustained Akt and Erk signaling, further enhancing proliferation, migration, and invasion. Consequently, the reciprocal regulation between *H19* and oncogenic signaling establishes a self-reinforcing circuit that amplifies EMT-driving transcriptional programs and supports tumor progression.

### 2.3. Epigenetic and Translational Regulation

*H19* has been shown to directly interact with the Polycomb Repressive Complex 2 (PRC2) component EZH2 in bladder cancer and tongue squamous cell carcinoma [52,53]. This interaction facilitates EZH2-mediated repression of epithelial markers, notably E-cadherin and ZO-1, while promoting expression of mesenchymal markers, including N-cadherin, vimentin, Snail1, Twist1, and ZEB1. Furthermore, the *H19*-EZH2 complex enhances Wnt/β-catenin signaling, thereby promoting EMT as well as tumor cell proliferation, invasion, and metastasis both in vitro and in vivo. Interestingly, Zhou et al. reported that a specific *H19* splicing variant, *H19*-L, which contains an alternative splicing between exon 1 and exon 4, is overexpressed in oral cancer [54]. *H19*-L binds ZEB1 mRNA, stabilizing it and increasing its translation, thereby inducing EMT and enhancing cell migration and proliferation. *H19* has also been shown to promote EMT and colorectal cancer metastasis through direct interaction with the heterogeneous nuclear ribonucleoprotein A2B1 (hnRNPA2B1) [14]. The *H19*-hnRNPA2B1 complex stabilizes Raf1 mRNA, leading to activation of the Raf-ERK signaling pathway. Notably, hnRNPA2B1 has also been reported to regulate EMT by preventing proteasomal degradation of ZEB1 [55]. Finally, *H19* has also been implicated in the regulation of alternative splicing programs through interactions with RNA-binding proteins. Recent studies have shown that *H19* can modulate splice variant expression, thereby influencing gene expression outputs in cancer cells [56,57]. However, whether this function contributes directly to EMT-MET regulation remains to be established. Given the emerging role of alternative splicing in EMT and MET regulation, this represents an important area for future investigation.

## 3. Role of miR-675 in Promoting Metastasis Through Both EMT and MET

While *H19* is widely recognized for promoting EMT and early metastatic dissemination, its derivative microRNA, miR-675, displays a context-dependent role. Processed from *H19*, both strands of miR-675 (miR-675-5p and miR-675-3p) are functional [19]. Notably, miR-675 can either promote or reverse EMT depending on the cellular context, suggesting a dual function in initiating EMT and facilitating MET. In the following section, we discuss how miR-675 modulates the EMT-MET continuum through regulation of transcription factors, signaling pathways, and cancer-related phenotypes (Table 2).

Similar to *H19*, miR-675 has been reported to promote EMT in various cancers (Table 2). In colorectal cancer, particularly during hypoxia-induced EMT, miR-675-5p upregulates Snail1 by targeting DDB2, a known repressor of Snail1 as well as other EMT-related genes, including VEGF and ZEB1 [62]. miR-675-5p also stabilizes HIF-1α, a master regulator of hypoxia-responsive genes. Clinically, elevated miR-675-5p levels correlate with lymph node metastasis, highlighting its potential as a marker of aggressiveness and metastatic dissemination. In contrast, in prostate cancer, the *H19*/miR-675 axis inhibits cell migration, a key EMT-related phenotype. miR-675 directly targets TGFBI, a pro-migratory extracellular matrix protein, reducing integrin-mediated adhesion and motility, thereby impairing EMT and metastatic potential [63]. In esophageal squamous cell carcinoma (ESCC), miR-675-3p promotes EMT and tumor aggressiveness. Its inhibition increases E-cadherin and decreases MMP-2 and MMP-9, supporting a role in EMT and metastatic dissemination [58]. Similarly, in osteosarcoma, exosomal miR-675 derived from highly metastatic cells induces EMT-like phenotypes in recipient osteoblasts by downregulating CALN1, enhancing migration and invasion. Clinically, patients with pulmonary metastases exhibit higher circulating exosomal miR-675 levels and lower CALN1 expression [64]. In cutaneous squamous cell carcinoma, miR-675 promotes EMT by targeting the tumor suppressor p53, leading to increased N-cadherin and vimentin and decreased E-cadherin expression [53]. Additionally, in ESCC, miR-675-5p enhances proliferation, migration, and invasion by targeting REPS2, a tumor suppressor that regulates RAC1 and CDC42 activity. This axis activates downstream effectors including MMP2, MMP9, and Cyclin D1, promoting EMT and tumor dissemination [60].

In contrast to the predominantly pro-EMT effects of *H19*, miR-675 displays a context-dependent role in modulating cancer cell phenotypes, with several studies highlighting a pro-MET (mesenchymal-to-epithelial transition) function in various cancers (Table 2). In hepatocellular carcinoma (HCC), miR-675 inhibits EMT and promotes MET in AFP-secreting HCC cell lines by targeting Twist1 and RB [61]. miR-675 overexpression upregulates E-cadherin and downregulates vimentin. Notably, forced expression of miR-675 in Sk-Hep cells, which display a spindle-shaped fibroblast-like morphology, induces epithelioid transformation and cellular clustering, accompanied by enhanced cell growth and reduced motility. Thus, while miR-675 suppresses EMT and invasion, it simultaneously promotes anchorage-independent growth. Similarly, in pancreatic cancer, miR-675-5p inhibits migration and invasion and promotes MET, as evidenced by increased E-cadherin and decreased N-cadherin, vimentin, Snail, Slug, and ZEB1 [59]. Mechanistically, miR-675-5p targets UBQLN1 (ubiquilin-1), a protein that sustains EMT transcription factor expression. UBQLN1 repression downregulates ZEB1 and disrupts the ZEB1/miR-200 feedback loop, thereby favoring MET. In non-small cell lung cancer (NSCLC), miR-675-5p promotes MET through inhibition of GPR55, a G protein-coupled receptor involved in oncogenic signaling [65]. Overexpression of miR-675-5p or GPR55 silencing reduces cell migration and invasion, whereas GPR55 restoration reverses these effects. GPR55 normally promotes EMT via ERK phosphorylation, RhoA-GTP signaling, Cyclin D1 expression, and MMP2/MMP9 induction. Consequently, miR-675-5p represses EMT and facilitates MET and metastatic colonization through GPR55 inhibition.

Beyond cancer, miR-675 can regulate EMT-related signaling pathways to promote MET. In ovarian cancer, both miR-675-5p and miR-675-3p target TGFβ1 and TGFBR2, resulting in downregulation of mesenchymal markers (N-cadherin, vimentin, Snail2) and upregulation of epithelial markers (E-cadherin, Cytokeratin-7) [66]. Repression of TGFβ1 and TGFBR2 inhibits SMAD2 signaling, reversing TGF-β-induced EMT and promoting epithelial reprogramming. A similar mechanism occurs in murine cardiac fibroblasts, where miR-675-5p and miR-675-3p target the 5’UTR and coding regions of TGFβR1, respectively, attenuating TGF-β signaling [67]. Additionally, miR-675 directly targets BMP pathway transcription factors SMAD1 and SMAD5 during skeletal muscle differentiation, suppressing EMT-promoting signals.

Collectively, these studies underscore the context-dependent dual function of miR-675 in regulating cellular phenotypes and tumor progression, capable of promoting either EMT or MET depending on tissue type, cellular context, and stage of cancer development.

## 4. Conclusions and Perspectives

The *H19*/miR-675 axis constitutes a central and dynamic regulator of epithelial–mesenchymal plasticity in cancer, coordinating EMT-driven dissemination and MET-associated colonization. Rather than acting as a linear driver, the H19/miR-675 axis operates within a highly interconnected regulatory network governing EMT/MET plasticity. Its context-dependent functions position this axis as a promising biomarker and a potential therapeutic target to limit metastatic progression.

**Figure 3:**
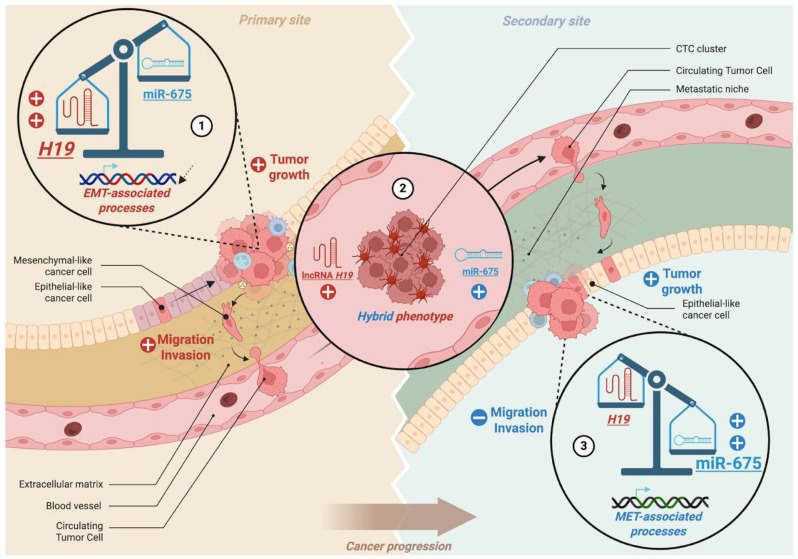
**Schematic illustration of the complementary roles of *H19* and its derivative miR-675 in cancer progression through EMT and MET regulation.** (1) At the primary tumor site, *H19* promotes EMT-associated processes, enhancing migration, invasion, and tumor growth. (2) The dynamic interplay between *H19* and miR-675 contributes to the acquisition of a hybrid epithelial/mesenchymal phenotype, favoring circulating tumor cell (CTC) cluster formation and metastatic dissemination. (3) At secondary sites, miR-675 promotes MET-associated programs, reducing migration and invasion, while supporting epithelial-like features and tumor colonization.

While fully mesenchymal cells often lack efficient re-epithelialization capacity and fully epithelial cells are poorly motile, transitional states with a hybrid epithelial/mesenchymal (E/M) phenotype exhibit enhanced adaptability, stemness, and metastatic potential [4,68,69]. Within this framework, *H19* and miR-675 may cooperate to favor metastasis: *H19* primarily drives EMT at the invasive front of primary tumors to facilitate cell detachment, whereas miR-675 may sustain a hybrid E/M phenotype, particularly in circulating tumor cells (CTCs), to promote extravasation (Figure 3). Supporting this hypothesis, Peperstraete et al. demonstrated that *H19* overexpression increases mesenchymal marker expression and decreases epithelial marker expression in triple-negative breast cancer cells, whereas miR-675 overexpression induces a more ambiguous phenotype, simultaneously upregulating epithelial and mesenchymal markers, indicative of a hybrid E/M state [13].

Interestingly, several studies have highlighted the pivotal role of the hybrid E/M phenotype in facilitating circulating tumor cell (CTC) dissemination, particularly through the formation of CTC clusters, typically composed of 2 to 50 cells, which detach collectively from the primary tumor and enter the bloodstream as cohesive units [70,71]. These cohesive CTC clusters exhibit enhanced resistance to anoikis and immune surveillance and display higher metastatic potential compared to single CTCs. Zhou et al. reported that *H19* is markedly upregulated in CTCs compared to primary tumors [29]. Mechanistically, *H19* modulates GIT2 and CYTH3, downstream effectors of the ARF GTPase pathway, which regulate cytoskeletal remodeling and cell motility. This axis sustains a hybrid E/M phenotype, promoting epithelial plasticity, resistance to anoikis, and survival of CTCs in circulation, thereby facilitating metastatic colonization. Genetic knockdown of *H19* or disruption of this pathway significantly reduces metastatic burden in syngeneic mouse models. Importantly, *H19* expression correlates with metastatic burden in human patients, supporting its potential as a biomarker of hematogenous dissemination and a therapeutic target for preventing metastasis.

From a clinical perspective, *H19* and miR-675 exhibit promising potential as diagnostic and prognostic biomarkers across multiple cancer types, including breast, colorectal, gastric, liver, pancreatic, bladder, ovary and esophageal cancers. However, their association with advanced tumor stages suggests that they may be more relevant as prognostic or progression-associated biomarkers rather than early diagnostic markers. Meta-analyses and transcriptomic studies have demonstrated that elevated levels of *H19* and miR-675 are strongly associated with advanced tumor stage, metastasis, and enrichment of EMT-related gene signatures, highlighting their value as progression-associated biomarkers, mesenchymal-like cancer phenotypes [12,72]. These findings not only reinforce the link between *H19* expression and EMT activation in patients but also suggest that *H19* and miR-675 could serve as non-invasive prognostic biomarkers, particularly in the context of liquid biopsies targeting CTCs or exosomes.

With growing insights into the dynamic regulation of EMT, modulation of the *H19*/miR-675 axis may represent a promising strategy to influence tumor cell plasticity. However, given the complexity of the regulatory networks involved in metastasis, its therapeutic potential is likely to be context-dependent and requires further validation. Supporting this approach, certain agents have demonstrated EMT-reversing effects. For example, ginsenoside Rd inhibits migration and invasion in tongue cancer cells by modulating the *H19*/miR-675-5p/CDH1 axis [73]. Another notable strategy is the plasmid-based gene therapy BC-819/*H19*-DTA, designed to drive diphtheria toxin A expression under the control of the *H19* promoter in platinum-resistant ovarian cancer [74]. Although this therapy does not directly inhibit *H19*, its tumor-specific expression allows selective cytotoxicity in malignant cells, demonstrating promising tolerability and disease stabilization in 31% of patients during a Phase I/IIa trial at Hebrew University.

On the miRNA front, strategies aimed at either restoring or inhibiting miR-675 represent a promising yet largely unexplored avenue. Although no clinical trials have specifically targeted miR-675 to date, the feasibility of miRNA-based therapeutics is supported by recent technological advances. For example, Lin et al. developed dual-peptide nanocarriers for miRNA delivery in cutaneous squamous cell carcinoma, while Hong et al. engineered elastin-like nanoparticles that improved the stability, circulation half-life, and tumor-targeting efficiency of therapeutic miRNAs [75,76]. These advances highlight the potential of nanocarrier-based approaches to modulate miR-675 activity and, thereby, influence EMT/MET plasticity in cancer. However, the involvement of miR-675 in physiological processes beyond cancer raises potential concerns regarding off-target effects and systemic toxicity. This highlights the need for tumor-specific delivery strategies and careful evaluation of therapeutic windows.

However, several challenges must be addressed before these strategies can be translated into clinical practice. First, the expression patterns and biological functions of *H19* and miR-675 are highly context-dependent, varying across tumor types, disease stages, and microenvironmental conditions. Second, accumulating evidence, including our own findings, indicates that neither *H19* nor miR-675 acts as a unidirectional driver of a fixed EMT or MET phenotype. Instead, their expression appears to reflect a transient, plastic state rather than a stable EMT or MET signature, limiting their potential as standalone biomarkers. Third, preclinical evidence remains sparse: most data derive from murine models, established cell lines, or retrospective analyses. Robust prospective clinical studies and standardized quantification methodologies are therefore required to define their prognostic or predictive value, particularly regarding metastatic prevention.

## Figures and Tables

**Table 1:** List of microRNAs directly regulated by *H19* and their validated EMT-associated targets in various cancers.

miRNA	Target	Cancer Type	Ref.
miR-17	KLF11	Breast cancer	[20]
	STAT3		
miR-22	MMP14	Liver cancer	[21]
	Snail1		
	Twist1	Osteosarcoma	[22]
	Snail1	Gastric cancer	[23]
miR-29	ETS1	Breast cancer	[20]
	TGFBR2		
	STAT3	Lung cancer	[24]
	PGRN	Colorectal cancer	[25]
	DNMT3B	Bladder cancer	[26]
miR-138	Vimentin	Colorectal cancer	[27]
	Zeb1		
	Zeb2		
miR-141	Zeb1	Gastric cancer	[28]
miR-200	Git2	Breast cancer	[29]
	SP1		[20]
	TGFBR2		
	ETS1		
	Zeb1	Colorectal cancer	[20]
	Zeb2		[27]
	CDK6	Glioma	[30]
	Zeb1		
miR-326	Twist1	Liver cancer	[31]
miR-340	miR-22	Breast cancer	[32]
miR-372/373	CXCR4	Cholangiocarcinoma	[33]
Let-7	HMGA2	Gynecologic cancer	[34]
	C-Myc		
	IMP3		
	STAT3	Esophageal cancer	[35]
	Lin28	Breast cancer	[35]
	Cyth3		[29]
	SP1		[20]
	TGFBR2		
	IL-6	Cholangiocarcinoma	[33]
	HMGA2	Pancreatic cancer	[36]

**Table 2:** Molecular targets of miR-675 and their functional implications in cancer progression.

Cancer Type	Molecular Actor Regulated by miR-675	Function of Mediator	Ref.
Esophageal cancer	REPS2	Increases cell proliferation, migration, invasion	[58]
Breast cancer	C-cbl	Increases cell proliferation, migration, invasion and survival	[51]
	Cbl-b	Increases cell proliferation, migration, invasion and survival	[51]
	Snail	Increases cell proliferation, migration, invasion, resistance and stemness	[11]
Lung cancer	GPR55	Increases cell proliferation, migration and invasion	[59]
Liver cancer	Twist1	Increases cell proliferation, migration and invasion	[60]
	RB	Increases cell proliferation	[60]
Pancreatic cancer	MMP2	Increases cell migration and invasion	[61]
	MMP9	Increases cell migration, invasion and angiogenesis	[61]
	Snail	Increases cell proliferation, migration, invasion, resistance and stemness	[61]
	UBQLN1	Increases cell migration, invasion and resistance	[61]
Gastric cancer	Snail	Increases cell proliferation, migration, invasion, resistance and stemness	[23]
	Snail	Increases cell proliferation, migration, invasion, resistance and stemness	[23]
Prostate cancer	TGF-β1	Increase cell migration, invasion and resistance	[62]
Skin cancer	p53	Decreases cell proliferation, migration, invasion and increases apoptosis	[52]
Bone cancer	CALN1	Increases cell migration and invasion	[63]

## Data Availability

No new data were created or analyzed in this study. Data sharing is not applicable to this article. All figures were created with biorender.com.

## References

[1] Brabletz T., Kalluri R., Nieto M.A., Weinberg R.A. (2018). EMT in cancer. Nat. Rev. Cancer.

[2] Prieto-García E., Díaz-García C.V., García-Ruiz I., Agulló-Ortuño M.T. (2017). Epithelial-to-mesenchymal transition in tumor progression. Med. Oncol..

[3] Kang E., Seo J., Yoon H., Cho S. (2021). The Post-Translational Regulation of Epithelial–Mesenchymal Transition-Inducing Transcription Factors in Cancer Metastasis. Int. J. Mol. Sci..

[4] Bakir B., Chiarella A.M., Pitarresi J.R., Rustgi A.K. (2020). EMT, MET, plasticity and tumor metastasis. Trends Cell Biol..

[5] Kalluri R., Weinberg R.A. (2009). The basics of epithelial-mesenchymal transition. J. Clin. Investig..

[6] Yilmaz M., Christofori G. (2009). EMT, the cytoskeleton, and cancer cell invasion. Cancer Metastasis Rev..

[7] Ocaña O.H., Corcoles R., Fabra A., Moreno-Bueno G., Acloque H., Vega S., Barrallo-Gimeno A., Cano A., Nieto M.A. (2012). Metastatic Colonization Requires the Repression of the Epithelial-Mesenchymal Transition Inducer Prrx1. Cancer Cell.

[8] Jehanno C., Vulin M., Richina V., Richina F., Bentires-Alj M. (2022). Phenotypic plasticity during metastatic colonization. Trends Cell Biol..

[9] Brannan C.I., Dees E.C., Ingram R.S., Tilghman S.M. (1990). The product of the H19 gene may function as an RNA. Mol. Cell. Biol..

[10] Collette J., Le Bourhis X., Adriaenssens E. (2017). Regulation of Human Breast Cancer by the Long Non-Coding RNA H19. Int. J. Mol. Sci..

[11] Lecerf C., Peperstraete E., Le Bourhis X., Adriaenssens E. (2020). Propagation and Maintenance of Cancer Stem Cells: A Major Influence of the Long Non-Coding RNA H19. Cells.

[12] Jing W., Zhu M., Zhang X.W., Pan Z.Y., Gao S.S., Zhou H., Qiu S.L., Liang C.Z., Tu J.C. (2016). The Significance of Long Noncoding RNA H19 in Predicting Progression and Metastasis of Cancers: A Meta-Analysis. BioMed Res. Int..

[13] Peperstraete E., Lecerf C., Collette J., Vennin C., Raby L., Völkel P., Angrand P.O., Winter M., Bertucci F., Finetti P. (2020). Enhancement of Breast Cancer Cell Aggressiveness by lncRNA H19 and its Mir-675 Derivative: Insight into Shared and Different Actions. Cancers.

[14] Zhang Y., Huang W., Yuan Y., Li J., Wu J., Yu J., He Y., Wei Z., Zhang C. (2020). Long non-coding RNA H19 promotes colorectal cancer metastasis via binding to hnRNPA2B1. J. Exp. Clin. Cancer Res..

[15] Zhao C.-C., Guo H., Wang Y., Li J.-H. (2021). Comprehensive upstream and downstream regulatory analyses identify miR-675-3p as a potential prognostic biomarker in melanoma. Hum. Cell.

[16] Ghafouri-Fard S., Esmaeili M., Taheri M. (2020). H19 lncRNA: Roles in tumorigenesis. Biomed. Pharmacother..

[17] Li T., Mo X., Fu L., Xiao B., Guo J. (2016). Molecular mechanisms of long noncoding RNAs on gastric cancer. Oncotarget.

[18] Angrand P.-O., Vennin C., Le Bourhis X., Adriaenssens E. (2015). The role of long non-coding RNAs in genome formatting and expression. Front. Genet..

[19] Cai X., Cullen B.R. (2007). The imprinted H19 noncoding RNA is a primary microRNA precursor. RNA.

[20] Li A., Mallik S., Luo H., Jia P., Lee D.F., Zhao Z. (2020). H19, a Long Non-coding RNA, Mediates Transcription Factors and Target Genes through Interference of MicroRNAs in Pan-Cancer. Mol. Ther. Nucleic Acids.

[21] Li L., Han T., Liu K., Lei C.G., Wang Z.C., Shi G.J. (2019). LncRNA H19 promotes the development of hepatitis B related hepatocellular carcinoma through regulating microRNA-22 via EMT pathway. Eur. Rev. Med. Pharmacol. Sci..

[22] Zhu S.-T., Wang X., Wang J.-Y., Xi G.-H., Liu Y. (2020). Downregulation of miR-22 Contributes to Epithelial-Mesenchymal Transition in Osteosarcoma by Targeting Twist1. Front. Oncol..

[23] Gan L., Lv L., Liao S. (2019). Long non-coding RNA H19 regulates cell growth and metastasis via the miR-22-3p/Snail1 axis in gastric cancer. Int. J. Oncol..

[24] Liu L., Liu L., Lu S. (2019). lncRNA H19 promotes viability and epithelial-mesenchymal transition of lung adenocarcinoma cells by targeting miR-29b-3p and modifying STAT3. Int. J. Oncol..

[25] Ding D., Li C., Zhao T., Li D., Yang L., Zhang B. (2018). LncRNA H19/miR-29b-3p/PGRN Axis Promoted Epithelial-Mesenchymal Transition of Colorectal Cancer Cells by Acting on Wnt Signaling. Mol. Cells.

[26] Lv M., Zhong Z., Huang M., Tian Q., Jiang R., Chen J. (2017). lncRNA H19 regulates epithelial-mesenchymal transition and metastasis of bladder cancer by miR-29b-3p as competing endogenous RNA. Biochim. Biophys. Acta Mol. Cell Res..

[27] Liang W.C., Fu W.M., Wong C.W., Wang Y., Wang W.M., Hu G.X., Zhang L., Xiao L.J., Wan D.C.C., Zhang J.F. (2015). The lncRNA H19 promotes epithelial to mesenchymal transition by functioning as miRNA sponges in colorectal cancer. Oncotarget.

[28] Zhou X., Ye F., Yin C., Zhuang Y., Yue G., Zhang G. (2015). The Interaction Between MiR-141 and lncRNA-H19 in Regulating Cell Proliferation and Migration in Gastric Cancer. Cell. Physiol. Biochem..

[29] Zhou W., Ye X.L., Xu J., Cao M.G., Fang Z.Y., Li L.Y., Guan G.H., Liu Q., Qian Y.H., Xie D. (2017). The lncRNA H19 mediates breast cancer cell plasticity during EMT and MET plasticity by differentially sponging miR-200b/c and let-7b. Sci. Signal..

[30] Chen X., Li Y., Zuo C., Zhang K., Lei X., Wang J., Yang Y., Zhang J., Ma K., Wang S. (2021). Long Non-Coding RNA H19 Regulates Glioma Cell Growth and Metastasis via miR-200a-Mediated CDK6 and ZEB1 Expression. Front. Oncol..

[31] Wei L.Q., Li L., Lu C., Liu J., Chen Y., Wu H. (2019). Involvement of H19/miR-326 axis in hepatocellular carcinoma development through modulating TWIST1. J. Cell. Physiol..

[32] Yan L., Yang S., Yue C.X., Wei X.Y., Peng W., Dong Z.Y., Xu H.N., Chen S.L., Wang W.R., Chen C.J. (2020). Long noncoding RNA H19 acts as a miR-340-3p sponge to promote epithelial-mesenchymal transition by regulating YWHAZ expression in paclitaxel-resistant breast cancer cells. Environ. Toxicol..

[33] Wang W.T., Ye H., Wei P.P., Han B.W., He B., Chen Z.H., Chen Y.Q. (2016). LncRNAs H19 and HULC, activated by oxidative stress, promote cell migration and invasion in cholangiocarcinoma through a ceRNA manner. J. Hematol. Oncol..

[34] Yan L., Zhou J., Gao Y., Ghazal S., Lu L., Bellone S., Yang Y., Liu N., Zhao X., Santin A.D. (2015). Regulation of tumor cell migration and invasion by the H19/let-7 axis is antagonized by metformin-induced DNA methylation. Oncogene.

[35] Chen M.J., Deng J., Chen C., Hu W., Yuan Y.C., Xia Z.K. (2019). LncRNA H19 promotes epithelial mesenchymal transition and metastasis of esophageal cancer via STAT3/EZH2 axis. Int. J. Biochem. Cell Biol..

[36] Ma C., Nong K., Zhu H., Wang W., Huang X., Yuan Z., Ai K. (2014). H19 promotes pancreatic cancer metastasis by derepressing let-7’s suppression on its target HMGA2-mediated EMT. Tumour Biol..

[37] Zhao Y., Feng C., Li Y., Ma Y., Cai R. (2019). LncRNA H19 promotes lung cancer proliferation and metastasis by inhibiting miR-200a function. Mol. Cell. Biochem..

[38] Xiong H., Shen J., Chen Z., Yang J., Xie B., Jia Y., Jayasinghe U., Wang J., Zhao W., Xie S. (2020). H19/let-7/Lin28 ceRNA network mediates autophagy inhibiting epithelial-mesenchymal transition in breast cancer. Int. J. Oncol..

[39] Viswanathan S.R., Powers J.T., Einhorn W., Hoshida Y., Ng T.L., Toffanin S., O’Sullivan M., Lu J., Phillips L.A., Lockhart V.L. (2009). Lin28 promotes transformation and is associated with advanced human malignancies. Nat. Genet..

[40] Kou N., Liu S., Li X., Li W., Zhong W., Gui L., Chai S., Ren X., Na R., Zeng T. (2019). H19 Facilitates Tongue Squamous Cell Carcinoma Migration and Invasion via Sponging miR-let-7. Oncol. Res..

[41] Morishita A., Zaidi M.R., Mitoro A., Sankarasharma D., Szabolcs M., Okada Y., D’Armiento J., Chada K. (2013). HMGA2 is a driver of tumor metastasis. Cancer Res..

[42] Dittmer J. (2015). The role of the transcription factor Ets1 in carcinoma. Semin. Cancer Biol..

[43] Chen L.-H., Hsu W.-L., Tseng Y.-J., Liu D.-W., Weng C.-F. (2016). Involvement of DNMT 3B promotes epithelial-mesenchymal transition and gene expression profile of invasive head and neck squamous cell carcinomas cell lines. BMC Cancer.

[44] Li J., Huang Y., Deng X., Luo M., Wang X., Hu H., Liu C., Zhong M. (2018). Long noncoding RNA H19 promotes transforming growth factor-β-induced epithelial-mesenchymal transition by acting as a competing endogenous RNA of miR-370-3p in ovarian cancer cells. OncoTargets Ther..

[45] Zhu J., Luo Z., Pan Y., Zheng W., Li W., Zhang Z., Xiong P., Xu D., Du M., Wang B. (2019). H19/miR-148a/USP4 axis facilitates liver fibrosis by enhancing TGF-β signaling in both hepatic stellate cells and hepatocytes. J. Cell. Physiol..

[46] Liu J., Wang G., Zhao J., Liu X., Zhang K., Gong G., Pan H., Jiang Z. (2022). LncRNA H19 Promoted the Epithelial to Mesenchymal Transition and Metastasis in Gastric Cancer via Activating Wnt/β-Catenin Signaling. Dig. Dis..

[47] Gao H., Hao G., Sun Y., Li L., Wang Y. (2018). Long noncoding RNA H19 mediated the chemosensitivity of breast cancer cells via Wnt pathway and EMT process. OncoTargets Ther..

[48] Zhang Y., Yan J., Li C., Wang X., Dong Y., Shen X., Zhang X. (2019). LncRNA H19 induced by helicobacter pylori infection promotes gastric cancer cell growth via enhancing NF-κB-induced inflammation. J. Inflamm..

[49] Zhao J., Ma S.-T. (2018). Downregulation of lncRNA H19 inhibits migration and invasion of human osteosarcoma through the NF-κB pathway. Mol. Med. Rep..

[50] Adriaenssens E., Lottin S., Berteaux N., Hornez L., Fauquette W., Fafeur V., Peyrat J.P., Le Bourhis X., Hondermarck H., Coll J. (2002). Cross-talk between mesenchyme and epithelium increases H19 gene expression during scattering and morphogenesis of epithelial cells. Exp. Cell Res..

[51] Vennin C., Spruyt N., Dahmani F., Julien S., Bertucci F., Finetti P., Chassat T., Bourette R.P., Le Bourhis X., Adriaenssens E. (2015). H19 non coding RNA-derived miR-675 enhances tumorigenesis and metastasis of breast cancer cells by downregulating c-Cbl and Cbl-b. Oncotarget.

[52] Luo M., Li Z., Wang W., Zeng Y., Liu Z., Qiu J. (2013). Long non-coding RNA H19 increases bladder cancer metastasis by associating with EZH2 and inhibiting E-cadherin expression. Cancer Lett..

[53] Zhang D.M., Lin Z.Y., Yang Z.H., Wang Y.Y., Wan D., Zhong J.L., Zhuang P.L., Huang Z.Q., Zhou B., Chen W.L. (2017). IncRNA H19 promotes tongue squamous cell carcinoma progression through β-catenin/GSK3β/EMT signaling via association with EZH2. Am. J. Transl. Res..

[54] Zhou W., Wang X.-Z., Fang B.-M. (2022). A variant of H19 transcript regulates EMT and oral cancer progression. Oral Dis..

[55] Wu Y., Yang X., Chen Z., Tian L., Jiang G., Chen F., Li J., An P., Lu L., Luo N. (2019). m^6^A-induced lncRNA RP11 triggers the dissemination of colorectal cancer cells via upregulation of Zeb1. Mol. Cancer.

[56] Cordaro A., Barreca M.M., Zichittella C., Loria M., Anello D., Arena G., Sciaraffa N., Coronnello C., Pizzolanti G., Alessandro R. (2024). Regulatory role of lncH19 in RAC1 alternative splicing: Implication for RAC1B expression in colorectal cancer. J. Exp. Clin. Cancer Res..

[57] Loria M., Anello D., Cordaro A., Zichittella C., Fontana S., Alessandro R., Conigliaro A. (2026). Long non-coding RNA H19 transported by colorectal cancer small extracellular vesicles promotes alternative splicing in healthy hepatocytes: New insights on liver pre-metastatic niche formation. Cell Commun. Signal..

[58] Xiao Q., Chen T., Wu Y., Wu W., Xu Y., Gong Z., Chen S. (2018). MicroRNA-675-3p promotes esophageal squamous cell cancer cell migration and invasion. Mol. Med. Rep..

[59] Wang J., Zhang Y., Wei H., Zhang X., Wu Y., Gong A., Xia Y., Wang W., Xu M. (2017). The mir-675-5p regulates the progression and development of pancreatic cancer via the UBQLN1-ZEB1-mir200 axis. Oncotarget.

[60] Zhou Y.W., Zhang H., Duan C.J., Gao Y., Cheng Y.D., He D., Li R., Zhang C.F. (2016). miR-675-5p enhances tumorigenesis and metastasis of esophageal squamous cell carcinoma by targeting REPS2. Oncotarget.

[61] Hernandez J.M., Elahi A., Clark C.W., Wang J., Humphries L.A., Centeno B., Bloom G., Fuchs B.C., Yeatman T., Shibata D. (2013). miR-675 Mediates Downregulation of Twist1 and Rb in AFP-Secreting Hepatocellular Carcinoma. Ann. Surg. Oncol..

[62] Costa V., Dico A.L., Rizzo A., Rajata F., Tripodi M., Alessand R., Conigliaro A. (2017). MiR-675-5p supports hypoxia induced epithelial to mesenchymal transition in colon cancer cells. Oncotarget.

[63] Zhu M., Chen Q., Liu X., Sun Q., Zhao X., Deng R., Wang Y., Huang J., Xu M., Yan J. (2014). lncRNA H19/miR-675 axis represses prostate cancer metastasis by targeting TGFBI. FEBS J..

[64] Gong L., Bao Q., Hu C., Wang J., Zhou Q., Wei L., Tong L., Zhang W., Shen Y. (2018). Exosomal miR-675 from metastatic osteosarcoma promotes cell migration and invasion by targeting CALN1. Biochem. Biophys. Res. Commun..

[65] He D., Wang J., Zhang C., Shan B., Deng X., Li B., Zhou Y., Chen W., Hong J., Gao Y. (2015). Down-regulation of miR-675-5p contributes to tumor progression and development by targeting pro-tumorigenic GPR55 in non-small cell lung cancer. Mol. Cancer.

[66] Zhao X., Liu Z., Li Y., Song S., Wang B., Pfeffer L.M., Zhang W., Yue J. (2025). MiR-675 Inhibits Primary Ovarian Tumor Growth and Metastasis by Suppressing EMT and TGFβ Signaling. J. Cancer.

[67] Dey B.K., Pfeifer K., Dutta A. (2014). The H19 long noncoding RNA gives rise to microRNAs miR-675-3p and miR-675-5p to promote skeletal muscle differentiation and regeneration. Genes Dev..

[68] Brabletz S., Schuhwerk H., Brabletz T., Stemmler M.P. (2021). Dynamic EMT: A multi-tool for tumor progression. EMBO J..

[69] Liao T.-T., Yang M.-H. (2020). Hybrid Epithelial/Mesenchymal State in Cancer Metastasis: Clinical Significance and Regulatory Mechanisms. Cells.

[70] Chen Q., Zou J., He Y., Pan Y., Yang G., Zhao H., Huang Y., Zhao Y., Wang A., Chen W. (2022). A narrative review of circulating tumor cells clusters: A key morphology of cancer cells in circulation promote hematogenous metastasis. Front. Oncol..

[71] Genna A., Vanwynsberghe A.M., Villard A.V., Pottier C., Ancel J., Polette M., Gilles C. (2020). EMT-Associated Heterogeneity in Circulating Tumor Cells: Sticky Friends on the Road to Metastasis. Cancers.

[72] Tsang W.P., Ng E.K., Ng S.S., Jin H., Yu J., Sung J.J., Kwok T.T. (2010). Oncofetal H19-derived miR-675 regulates tumor suppressor RB in human colorectal cancer. Carcinogenesis.

[73] Chang L., Wang D., Kan S., Hao M., Liu H., Yang Z., Xia Q., Liu W. (2022). Ginsenoside Rd inhibits migration and invasion of tongue cancer cells through H19/miR-675-5p/CDH1 axis. J. Appl. Oral Sci. Rev..

[74] Lavie O., Edelman D., Levy T., Fishman A., Hubert A., Segev Y., Raveh E., Gilon M., Hochberg A. (2017). A phase 1/2a, dose-escalation, safety, pharmacokinetic, and preliminary efficacy study of intraperitoneal administration of BC-819 (H19-DTA) in subjects with recurrent ovarian/peritoneal cancer. Arch. Gynecol. Obstet..

[75] Hong J., Sim D., Lee B.-H., Sarangthem V., Park R.-W. (2024). Multifunctional elastin-like polypeptide nanocarriers for efficient miRNA delivery in cancer therapy. J. Nanobiotechnol..

[76] Lin C.Y., Fang J.Y., Hsiao C.Y., Lee C.W., Alshetaili A., Lin Z.C. (2025). Dual cell-penetrating peptide-conjugated polymeric nanocarriers for miRNA-205-5p delivery in gene therapy of cutaneous squamous cell carcinoma. Acta Biomater..

